# Excessive visceral fat area as a risk factor for early postoperative complications of total gastrectomy for gastric cancer: a retrospective cohort study

**DOI:** 10.1186/s12893-016-0168-8

**Published:** 2016-08-05

**Authors:** Masashi Takeuchi, Kenjiro Ishii, Hiroaki Seki, Nobutaka Yasui, Michio Sakata, Akihiko Shimada, Hidetoshi Matsumoto

**Affiliations:** Department of Surgery, Keiyu Hospital, 3-7-3 Minatomirai, Nishi-ku, Yokohama-shi, Kanagawa 220-8521 Japan

**Keywords:** Excessive visceral fat area, Total gastrectomy, Gastric cancer, Anastomotic leakage, Incisional SSI

## Abstract

**Background:**

Obesity is a known risk factor for complications after digestive surgery. Body mass index (BMI) is commonly used as an index of obesity but does not always reflect the degree of obesity. Although some studies have shown that high visceral fat area (VFA) is associated with poor outcomes in digestive surgery, few have examined the relationship between VFA and total gastrectomy. In this study, we demonstrated that VFA is more useful than BMI in predicting complications after total gastrectomy.

**Methods:**

Seventy-five patients who underwent total gastrectomy for gastric cancer were enrolled in this study; they were divided into two groups: a high-VFA group (*n* = 26, ≥100 cm^2^) and a low-VFA group (*n* = 49, <100 cm^2^). We retrospectively evaluated the preoperative characteristics and surgical outcomes of all patients and examined postoperative complications within 30 days of surgery (including cardiac complications, pneumonia, ileus, anastomotic leakage, pancreatic fistula, incisional surgical site infection [SSI], abdominal abscess, and hemorrhage).

**Results:**

The incidence of anastomotic leakage (*p* = 0.03) and incisional SSI (*p* = 0.001) were higher in the high-VFA group than in the low-VFA group. No significant differences were observed in the other factors. We used univariate analysis to identify risk factors for anastomotic leakage and incisional SSI. Age and VFA were risk factors for anastomotic leakage, and BMI and VFA were risk factors for incisional SSI. A multivariate analysis including these factors found that only VFA was a predictor of anastomotic leakage (hazard ratio [HR] 4.62; 95 % confidence interval [CI] 1.02–21.02; *p* = 0.048) and incisional SSI (HR 4.32; 95 % CI 1.18–15.80; *p* = 0.027].

**Conclusions:**

High VFA is more useful than BMI in predicting anastomotic leakage and SSI after total gastrectomy. Therefore, we should consider the VFA value during surgery

## Background

Total gastrectomy for gastric cancer is one of the highly invasive surgeries in gastroenterology, and is associated with high morbidity and mortality. A recent study reported a 30-day morbidity rate of 36 % and mortality rate of 4.7 % after total gastrectomy [1], with common postoperative complications being respiratory complications (16 %), sepsis (15 %), organ/space infection (9 %), and surgical site infection (SSI) (8 %). Other known severe complications are pancreatic fistula and anastomotic insufficiency. As these are difficult to manage, careful management of postoperative complications in total gastrectomy is necessary.

Obesity is a known risk factor for postoperative complications in digestive surgery [2]. Although body mass index (BMI) is commonly used as an index of obesity, it does not always reflect the degree of obesity [3]. It has also been reported that Asians have a higher percentage of body fat than Caucasians at the same BMI level [4, 5].

Recent studies have shown that high visceral fat area (VFA) is associated with poor outcomes in digestive surgery [6, 7]. However, there have been few studies on the relationship between VFA and total gastrectomy.

In the present study, we demonstrated that VFA is more useful than BMI in predicting postoperative complications in total gastrectomy.

## Methods

### Patients

Seventy-five patients who underwent total gastrectomy for gastric cancer at the Keiyu Hospital, Kanagawa, Japan between June 2009 and February 2015. There was no limitation with regards to age, and patients’ ECOG performance status scores ranged from 0 to 2. Patients who underwent total gastrectomy with combined resection of other organs and those who had surgery following neoadjuvant chemotherapy were also included in the sample.

Patients’ preoperative examinations included upper gastrointestinal endoscopy, abdominal computed tomography (CT) scan, and laboratory tests. Gastric cancer diagnoses were based on pathologic findings [8].

Lymph node dissection and gastric reconstruction were determined according to the Japanese classification of gastric carcinoma [8]. Patients with clinical T2, T3, T4a, T4b, or N+ underwent D2 dissection; those with clinical T1a or a part of T1b with N0 underwent D1 or D1+ dissection. All patients underwent Roux-en-Y reconstruction. We resected the transverse colon or pancreas tail simultaneously if there was direct tumor invasion of both organs.

We retrospectively evaluated patients’ preoperative characteristics from hospital records, including age, sex, history of diabetes mellitus, cardiac history, pulmonary history, and history of chronic kidney disease. We also assessed intraoperative findings, such as gastrectomy with splenectomy, gastrectomy with jejunostomy, number of retrieved lymph nodes, operating time, amount of blood loss, and pathologic stage. Pathologic findings were defined by the Japanese classification of gastric carcinoma. We obtained BMI data and American Society of Anesthesiologists (ASA) scores from patients’ anesthesia records. Three patients who had surgery following neoadjuvant chemotherapy were included. Three patients received neoadjuvant chemotherapy preoperatively. S-1 was administered to one patient, and two patients received S-1 + cisplatin.

We collected data on postoperative complications within 30 days of surgery. This included cardiac complications, pneumonia, ileus, anastomotic leakage, pancreatic fistula, incisional SSI, abdominal abscess, and hemorrhage. Anastomotic leakage was diagnosed on the basis of CT scan findings or the characteristics of abdominal drains. Pancreatic fistula was diagnosed if the amylase content of the drain around the pancreas after postoperative day 3 was greater than three times the upper limit of its normal serum value [9]. The diagnosis of incisional SSI was based on the definition of the United States Centers for Disease Control and Prevention Guidelines for the prevention of SSIs [10]. We used the Clavien–Dindo classification for complications and identified complication cases as those having a Clavien–Dindo classification greater than grade 2, with the exception of incisional SSI cases. Incisional SSI was only investigated as postoperative SSI. The treatment for incisional SSI is open drainage of wound infections; this treatment represents a grade 1 Clavien–Dindo classification. Therefore, we selected patients with a Clavien–Dindo classification greater than grade 1 for incisional SSI.

We excluded one patient who underwent laparoscopic gastrectomy. Patients with esophagogastric junction cancer, those who underwent emergency surgery due to gastric perforation, and one patient who underwent additional total gastrectomy after a positive surgical margin post-distal gastrectomy were also excluded. This study was approved by the Keiyu hospital ethics committee (approval number:H27-No31).

### Evaluation of fat area

We measured VFA and subcutaneous fat area (SFA) at the umbilical level on available CT scan images (LightSpeed VCT 64 slice CT, GE Yokogawa Medical Systems). CT was performed 4 weeks preoperatively. To calculate VFA, we first traced the outline of the intraperitoneal tissue [11, 12]. Thereafter, using this outlined region, we determined a histogram of the CT numbers ranging from −150 HU to −50 HU [13]. SFA was calculated in a similar manner by using a manually traced contour of the subcutaneous region. Japanese criteria for obesity disease have been provided by the Japan Society for Study of Obesity [3]. These criteria were adopted by the Japanese Ministry of Health, Labour and Welfare and set the cut-off value of visceral obesity as 100 cm2. As only Japanese individuals were included in our study, we used a cut-off value of 100 cm2 for VFA. Patients were divided into two groups: a high-VFA group (*n* = 26, ≥100 cm^2^) and a low-VFA group (*n* = 49, <100 cm^2^).

### Statistical analysis

Statistical analyses were performed using Stata/SE 12.1 for Mac (StataCorp, TX, USA). Categorical variables were analyzed with chi-square tests for univariate analysis, and continuous variables were analyzed with the Mann–Whitney U test. A *p* value < 0.05 was considered significant. Variables with *p* values < 0.05 in the univariate analysis were subsequently entered into a logistic regression model for multivariate analysis.

## Results

### Comparison of baseline characteristics

We compared the baseline characteristics of the two groups (Table 1). The groups were similar in terms of mean age (high VFA group: 70.8 ± 9.7 years vs. low VFA group: 70.7 ± 10.2 years; *p* = 0.71), ASA scores, disease stage, and underlying diseases. The groups differed significantly on BMI (*p* < 0.0001), VFA (*p* < 0.0001), SFA (*p* < 0.0001), total fat area (*p* < 0.0001), and history of chronic kidney disease (*p* = 0.005).

**Table 1:** Comparison of the baseline characteristics of patients who underwent total gastrectomy (*n* = 75)

	High-VFA (*n* = 26)	Low-VFA (*n* = 49)	*p* value
Sex (men/women)	22/4	35/14	0.20
Age (mean ± SD)	70.8 ± 9.7	70.7 ± 10.2	0.71
ASA (I/II/III)	7/15/4	6/36/7	0.25
BMI (kg/m^2^) in mean ± SD	25.1 ± 3.0 (20.0–32.2)	20.8 ± 2.6 (14.7–25.4)	<0.0001
VFA (cm^2^) (mean ± SD)	146.9 ± 38.2 (101.8–240.9)	54.3 ± 27.1 (5.9–99.9)	<0.0001
SFA (cm^2^) (mean ± SD)	157.1 ± 54.8 (77.3–292.3)	86.1 ± 54.9 (5.1–200.5)	<0.0001
Total fat area (cm^2^) (mean ± SD)	304.0 ± 74.7 (194.7–481.2)	140.5 ± 72.0 (14.3–286.0)	<0.0001
Diabetes mellitus	7	7	0.18
Cardiac history	5	8	0.75
Pulmonary history	1	3	0.68
Chronic kidney disease	4	0	0.005
Pathologic T (1a/1b/2/3/4a/4b)	1/7/7/4/7/0	2/6/9/20/10/2	0.19
Pathologic N (0/1/2/3)	12/4/3/7	17/6/13/13	0.48
Pathologic M (0/1)	26/1	44/5	0.33
Pathologic stage (I/II/III/IV)	11/6/8/1	14/10/20/5	0.51
Neoadjuvant chemotherapy	0	3	0.198
Residual gastrectomy	0	6	0.06
Splenectomy	8	10	0.32
Resection of pancreatic tail	1	2	0.24
Partial resection of colon	1	1	0.64

*SD* standard deviation; *ASA* American Society of Anesthesiologists; *BMI* body mass index; *SFA* subcutaneous fat area; *VFA* visceral fat area; *T* tumor status; *N* nodal status; *M* metastasis status

### Comparison of surgical outcomes and postoperative complications

Compared with the low-VFA group, the high VFA group had a higher incidence of anastomotic leakage (*p* = 0.03) and incisional SSI (*p* = 0.001). No significant differences were observed for the other factors (Table 2).

**Table 2:** Comparison of outcomes and complications after total gastrectomy (*n* = 75)

	High-VFA (*n* = 26)	Low-VFA (*n* = 49)	*p* value
Number of retrieved lymph nodes (mean ± SD)	42.7 ± 18.7	43.0 ± 24.2	0.81
Operating time (min) in mean ± SD	225.9 ± 92.0	200.9 ± 58.3	0.08
Blood loss (ml) in mean ± SD	467.9 ± 600.2	318.5 ± 407.1	0.32
Postoperative hospital stay (day) in mean ± SD	22.8 ± 15.7	18.8 ± 9.6	0.70
Postoperative complications			
Cardiac	0	3	0.20
Pneumonia	4	4	0.33
Incisional SSI	12	6	0.001
Ileus	2	0	0.05
Anastomotic leakage	6	3	0.03
Pancreatic fistula	2	1	0.23
Abdominal abscess	4	3	0.19
Postoperative hemorrhage	1	2	0.96
Other	4	5	0.53

*SD* standard deviation; *SSI* surgical site infection; *VFA* visceral fat area

### Risk factors for anastomotic leakage and incisional SSI

We used univariate analysis to determine the risk factors for anastomotic leakage and incisional SSI from variables, such as background and surgical outcomes. Age and VFA were risk factors for anastomotic leakage, and BMI and VFA were risk factors for incisional SSI. In the multivariate analysis that included these factors, only VFA was identified as a predictor of anastomotic leakage (hazard ratio [HR] 4.62; 95 % confidence interval [CI] 1.02–21.02; *p* = 0.048] (Tables 3 and 4) and incisional SSI (HR 4.32; 95 % CI 1.18–15.80; *p* = 0.027) (Tables 5 and 6).

**Table 3:** Univariate analyses of factors associated with anastomotic leakage following total gastrectomy

Risk factors	anastomotic leakage (+)	anastomotic leakage (−)	Univariate analysis	Logistic regression analysis	
				Hazard ratio (95 % CI)	*p* value
Sex					
Men	9	48	0.072		
Women	0	18			
Age (mean ± SD)	64.6 ± 7.5	71.6 ± 10.0	0.017	0.94 (0.87–1.01)	0.057
ASA					
I	2	11	0.891		
II	6	45			
III	1	10			
BMI (≥25/<25 kg/m^2^)	4/5	12/54	0.071		
VFA (≥100/<100 cm^2^)	6/3	20/46	0.032	4.6 (1.05–20.25)	0.044
SFA (cm^2^) (mean ± SD)	126.5 ± 56.5	108.6 ± 65.3	0.434		
Diabetes mellitus	2	12	0.77		
Cardiac history	2	11	0.695		
Pulmonary history	0	4	0.444		
Chronic kidney disease	1	3	0.419		
Pathologic Stage					
I	1	24	0.269		
II	3	13			
III	5	23			
IV	0	6			
Neoadjuvant chemotherapy	0	3	0.514		
Residual gastrectomy	0	6	0.346		
Splenectomy	2	16	0.894		
Resection of pancreatic tail	1	2	0.246		
Partial resection of colon	0	2	0.597		
Number of retrieved lymph nodes (mean ± SD)	38.4 ± 22.3	43.5 ± 22.4	0.487		
Operating time (min) (mean ± SD)	254.8 ± 118.8	207.3 ± 59.0	0.23		
Blood loss (ml) (mean ± SD)	572.5 ± 663.7	351.4 ± 460.2	0.428		

*CI* confidence interval; *SD* standard deviation

**Table 4:** Multivariate analysis of factors associated with anastomotic leakage after total gastrectomy

Risk factors	anastomotic leakage (+)	anastomotic leakage (−)	β	SE	Hazard ratio (95 % CI)	*p* value
Age (≥70/<70 years)	2/7	35/31	−0.20	0.22	0.25 (0.05–1.36)	0.108
VFA (≥100/<100 cm^2^)	6/3	20/46	0.25	3.57	4.62 (1.02–21.02)	0.048

CI, confidence interval; β, standard regression coefficient; SE, standard error

**Table 5:** Univariate analyses of factors associated with incisional SSI following total gastrectomy

Risk factors	Incisional SSI (+)	Incisional SSI (−)	Univariate analysis	Logistic regression analysis	
				Hazard ratio (95 % CI)	*p* value
Sex					
Men	15	42	0.403		
Women	3	15			
Age (years)	71.2 ± 9.4	70.6 ± 10.2	0.98		
ASA					
I	1	12	0.318		
II	14	37			
III	3	8			
BMI (≥25/<25 kg/m^2^)	8/10	8/49	0.006	4.9 (1.49–16.15)	0.009
VFA (≥100/<100 cm^2^)	12/6	14/43	0.001	6.14 (1.94–19.41)	0.002
SFA (cm^2^) (mean ± SD)	136.9 ± 73.3	102.5 ± 59.5	0.104		
Diabetes mellitus	3	11	0.803		
Cardiac history	5	8	0.144		
Pulmonary history	1	3	0.921		
Chronic kidney disease	2	2	0.186		
Pathologic Stage					
I	6	19	0.527		
II	4	12			
III	8	20			
IV	0	6			
Neoadjuvant chemotherapy	1	2	0.699		
Residual gastrectomy	0	6	0.151		
Splenectomy	3	15	0.403		
Resection of pancreatic tail	1	2	0.699		
Partial resection of colon	1	1	0.383		
Number of retrieved lymph nodes (mean ± SD)	39.7 ± 18.2	43.9 ± 23.5	0.85		
Operating time (min) (mean ± SD)	225.1 ± 64.2	208.6 ± 69.6	0.278		
Blood loss (ml) (mean ± SD)	368.2 ± 369.9	377.4 ± 517.7	0.985		

*SSI* surgical site infection; *CI* confidence interval; *SD* standard deviation

**Table 6:** Multivariate analysis of factors associated with incisional SSI after total gastrectomy

Risk factors	Incisional SSI (+)	Incisional SSI (−)	β	SE	Hazard ratio (95 % CI)	*p* value
BMI (≥25/<25 kg/m^2^)	8/10	8/49	0.31	1.60	2.28 (0.57–9.04)	0.241
VFA (≥100/<100 cm^2^)	12/6	14/43	0.38	2.86	4.32 (1.18–15.80)	0.027

*CI* confidence interval; *β* standard regression coefficient; *SE* standard error

## Discussion

We reached two conclusions based on the results of our study: 1) high VFA is a more useful risk factor than high BMI in predicting anastomotic leakage after total gastrectomy, and 2) compared with high SFA, high VFA resulted in more incisional SSIs.

There have been some studies on the relationship between VFA and complications following digestive surgery [14, 15]. A few studies have reported that VFA was a more useful index than BMI in predicting postoperative complications in gastrectomy. Sugisawa et al. indicated that excessive visceral fat was an independent risk factor for pancreas-related infection and anastomotic leakage after gastrectomy [16]. Tokunaga et al. investigated the relationship between fat area and early surgical outcomes after gastrectomy [17] and concluded that excessive visceral fat was likely to result in intra-abdominal infections, such as anastomotic leakage, pancreas-related infection, and intra-abdominal abscess. Tanaka et al. evaluated risk factors (including VFA) for postoperative complications after total gastrectomy [18] and found that the VFA value was a better indicator of pancreatic fistula compared with BMI. Our study showed that VFA was useful in predicting anastomotic leakage. Previous studies did not consider background characteristics (e.g., cardiovascular diseases) that are usually associated with patients with obesity; these background factors may have contributed to the incidence of anastomotic leakage due to insufficient microcirculation [19, 20] and may have confounded their results. Therefore, in our study, we considered baseline characteristics, such as cardiac history or diabetes mellitus, which may affect the incidence of anastomotic leakage.

Kim et al. showed that male sex, preoperative/intraoperative transfusion, cardiovascular disease, and disease location on the upper third of the stomach were predictive of postoperative anastomotic leakage after gastrectomy [19]. Although some factors, such as splenectomy or malnutrition, were identified as risk factors for anastomotic leakage [21, 22], excessive tension on the anastomosis site was also reported to be a risk factor [16, 23].

In our study, high VFA resulted in more incisional SSIs compared with high SFA. Mike et al. evaluated the incidence of incisional SSI and identified the predictors after digestive surgery [24]. They identified four risk factors for incisional SSI after stoma reversal: history of fascial dehiscence, colostomy, Caucasian origin, and thick subcutaneous fat. In the present study, incisional SSI was observed in 18 patients, seven (39 %) of whom had anastomotic leakage. Therefore, anastomotic leakage was a confounding factor. Moreover, there are large confidence intervals for VFA in multivariate analysis, because our single-center study had a small number of patients. We thought that it was not appropriate to investigate the patients in more previous periods for increasing the number of patients, because patients who underwent a surgery of different quality might also be included.

This study had several limitations. First, it was a retrospective, single-center study limited to Asian population. Our hospital also has extensive experience and a high workload in gastric cancer surgery due to higher local incidence; thus, our outcomes may not be applicable to other centers in other countries. Second, to calculate VFA, the outlines of intraperitoneal tissue were traced manually; this may have led to measurement errors, compared with automatic tracing.

## Conclusions

High VFA is more useful than BMI in predicting anastomotic leakage and SSI after total gastrectomy. Therefore, we should consider the VFA value during surgery.
